# Hump-Nosed Viper Bite-Associated Thrombotic Thrombocytopenic Purpura: A Rare Complication

**DOI:** 10.7759/cureus.55873

**Published:** 2024-03-09

**Authors:** Manojkumar Krishnan, KVC Janaka, Hassan Hussain, Hiruni Fernando, Chitranga Kariyawasan

**Affiliations:** 1 Internal Medicine, Sri Jayewardenepura General Hospital, Colombo, LKA; 2 Hematology, Sri Jayewardenepura General Hospital, Colombo, LKA

**Keywords:** complications of snake bite, hump-nosed viper, acute kidney injury, plasmic score, therapeutic plasma exchange (tpe), thrombotic thrombocytopenic purpura, microangiopathic hemolytic anemia (maha)

## Abstract

Thrombotic thrombocytopenic purpura (TTP) is one of the rarely encountered complications of hump-nosed viper bites, which requires early detection and specific management. Hump-nosed viper bites are well known to affect multiple systems, and it is imperative to identify and manage each complication simultaneously.

A 48-year-old patient presented to the hospital following a hump-nosed viper bite, where he subsequently developed local necrosis, acute kidney injury (AKI), and TTP. A diagnosis of TTP was made using the PLASMIC score (which refers to the score’s seven components: platelet count; combined hemolysis variable; absence of active cancer; absence of stem-cell or solid organ transplant; mean corpuscular volume (MCV); international normalized ratio (INR); and creatinine) and supporting blood picture findings despite the diagnostic difficulties encountered due to the misleadingly normal automated platelet counts. The patient underwent multiple blood transfusions, 12 cycles of hemodialysis, and two cycles of therapeutic plasma exchange, the latter contributing to a significant improvement in his overall clinical and biochemical markers.

In this case presentation, we report a rare case of TTP occurring after a hump-nosed viper bite, with the outcome of the report focusing on the diagnostic difficulties and available therapeutic modalities.

## Introduction

Snakebites are a rather neglected clinical entity, leading to significant morbidity and mortality, especially in tropical countries. The hump-nosed viper is a highly venomous species, and bites can result in a plethora of reactions extending from local necrosis to a raft of systemic complications involving the hematological, neurological, renal, pulmonary, and cardiac systems. In this case report, we report a presentation of TTP, a recognized rare complication [1], occurring following a hump-nosed viper bite.

Thrombotic thrombocytopenic purpura (TTP) is a microangiopathic hemolytic anemia caused by an inherited or acquired deficiency of ADAMTS13 [1]. Among many others, hump-nosed viper bites are known to be associated with hematological complications such as venom-induced consumption coagulopathy (VICC); TTP can manifest as a part of VICC [2].

In this case report, we report a presentation of TTP, a recognized rare complication [3], occurring following a hump-nosed viper bite.

## Case presentation

**Figure 1:**
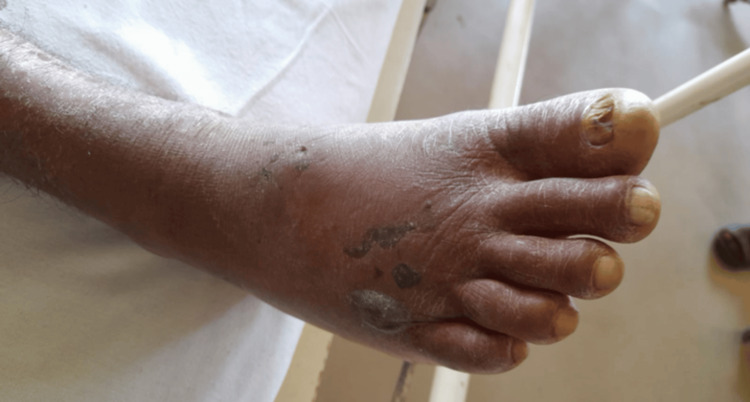
Localized swelling and the necrotic papules forming on the patient's foot following the hump-nosed viper bite

A previously healthy 48-year-old male presented to his local hospital with a history of hump-nosed viper bite on the dorsum of his right foot. The incident occurred two days earlier, but he applied local home remedies before getting admitted. On admission to the local hospital, he had features suggestive of acute renal failure (ARF) and marked localized swelling with a necrotic papule (Figure 1).

Initial investigations done at the local hospital revealed that he had a white blood cell (WBC) count of 16.44×10^3/µl with hemoglobin (Hb) of 9.1 g/dl and a platelet count of 150×10^3/µl. Serial serum creatinine levels had also been performed at the local hospital, which showed that his serum creatinine was high and rising from 7.12 mg/dL (629 µmol/L) to 7.54 (666 µmol/L) over the course of two days, with the diagnosis of acute kidney injury (AKI) further reinforced by the supportive findings in the ultrasound scan of the abdomen and pelvis. The rest of the blood investigations performed at the local hospital, including the clotting profile, liver functions, liver enzymes, and serum electrolytes, were within the normal ranges.

Three cycles of hemodialysis had been done at the local hospital, but his symptoms had not improved. Four days later, he was transferred to a tertiary care hospital for further management.

Upon admission to the ward, the patient exhibited varied symptoms, including fever, swelling in the right lower limb with a necrotic papule, reduced urine output, vomiting, loose stools, anuria, shortness of breath, and a persistent headache, but he did not exhibit any bleeding manifestations. During the examination, the patient was conscious and rational, with a blood pressure of 110/70 mmHg, a pulse rate of 98 beats per minute, an oxygen saturation (SpO_2_) level of 95% on room air, and a Glasgow coma scale (GCS) of 15 out of 15 with no focal neurological deficits. The patient's abdomen was soft and non-tender, while the chest examination revealed bilateral crepitations. His clinical syndrome was compatible with a hump-nosed viper bite. Considering the known but rare association with thrombotic thrombocytopenic microangiopathy, tests were sent, including a blood picture specifically to look for evidence of thrombotic microangiopathy and a PLASMIC score (which refers to the score’s seven components: platelet count; combined hemolysis variable; absence of active cancer; absence of stem-cell or solid organ transplant; mean corpuscular volume (MCV); international normalized ratio (INR); and creatinine). His initial investigation done at the tertiary care hospital showed that his Hb level had further dropped to 5.9 g/dL with a platelet count of 140×10^3/µl (automated machine count) (Table 1).

**Table 1:** Summary of the investigations performed on the patient WBC: white blood cells; Hb: hemoglobin; CRP: C-reactive protein; LDH: lactate dehydrogenase; AST: aspartate transaminase; ALT: alanine transaminase; and ALP: alkaline phosphatase; N/A: not applicable

Investigation (unit)	Day 1	Day 2	Day 3	Day 4	Day 5 (5.00 AM)	Day 5 (4.00 PM)	Day 6	Day 7	Reference range
WBC (×10^3/µL)	11.84	14.05	9.96	12.61	11.55	12.73	14.23	11.58	4 - 10
Hb (g/dL)	5.9	6.7	4.9	6.8	7.6	9.2	9.9	8.4	11 - 16
Platelets (×10^3/µL)	140	140	127	106	112	119	153	212	150 - 450
Manual platelet count (×10^3/µL)	N/A	N/A	25	N/A	62	100	135	182	150 - 450
CRP (mg/L)	6	N/A	97	N/A	94	N/A	57	N/A	<6
LDH (U/L)	N/A	N/A	454	N/A	1416	923	930	942	140 - 280
Serum creatinine (µmol/L)	738	545	610	N/A	538	839	692	953	45 - 90
Blood urea (mg/dL)	167	N/A	N/A	N/A	N/A	N/A	150	N/A	17 -45
Sodium (mmol/L)	134	138	137	N/A	138	138	139	N/A	136 - 145
Potassium (mmol/L)	4.1	4.1	4.3	N/A	4.2	4.3	3.9	N/A	3.5 – 5.3
AST (U/L)	179	N/A	N/A	N/A	N/A	N/A	24	N/A	0 – 31
ALT (U/L)	83	N/A	N/A	N/A	N/A	N/A	23	N/A	7 – 35
ALP (U/L)	50	N/A	N/A	N/A	N/A	N/A	50	N/A	30 -120
Total bilirubin (mg/dL)	2.6	N/A	N/A	N/A	N/A	N/A	1.6	N/A	0.2 – 1.1

As the patient had an AKI with a high serum creatinine of 738 µmol/L, he underwent hemodialysis with a two-pint blood transfusion. After the procedure, the patient's Hb level rose to 6.7 g/dL with a platelet count of 140×10^3/µl (automated machine count), and his serum creatinine value dropped to 545 µmol/L (Table 1). 

**Figure 2:**
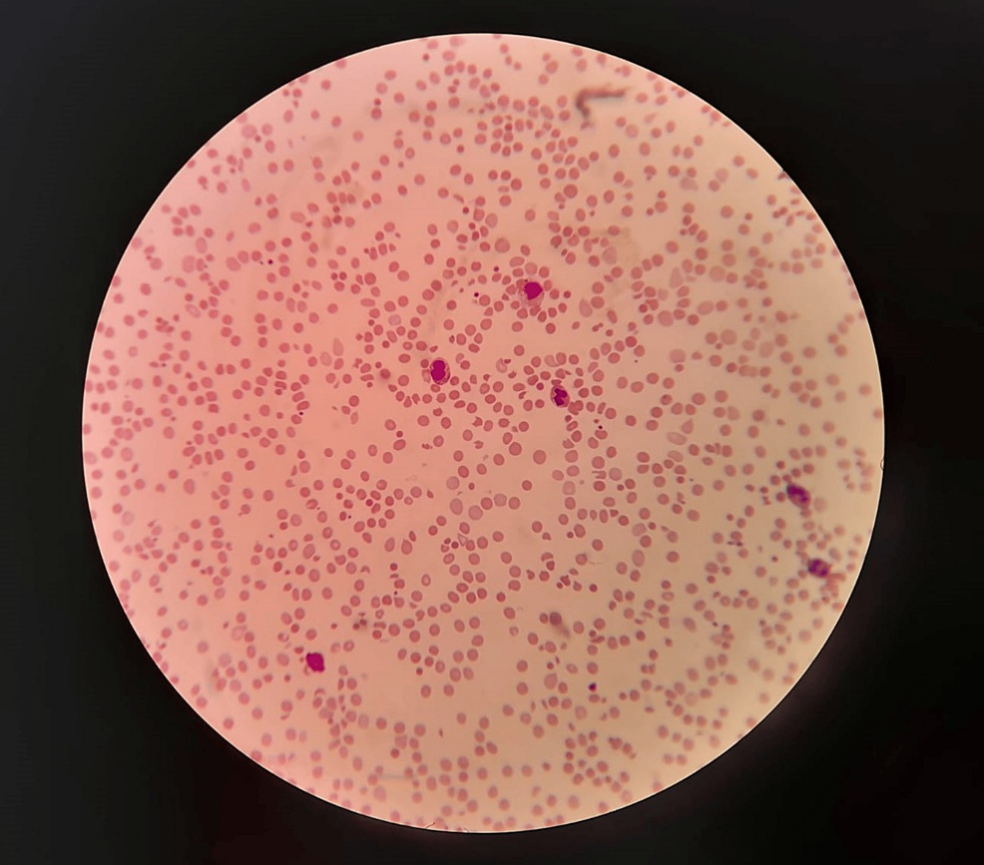
A blood picture of the 48-year-old male presenting following the hump-nosed viper bite

On day three since admission, upon examination, the patient's blood pressure was 118/68 mmHg, his pulse rate was 91 bpm, and his SpO_2_ was 97% on the non-rebreather mask (NRBM). He was confused, had bilateral basal crepitation on lung auscultation, and had tenderness in the upper abdomen. His blood reports revealed that his Hb had again dropped to 4.9 g/dL with a machine-generated platelet count of 127×10^3/µL and a visual platelet count of 25x10^3/µL (Table 1). His blood picture findings are presented in Table 2 and Figure 2.

**Table 2:** Blood picture findings of the 48-year-old male presenting following the hump-nosed viper bite

Cell type	Findings	Comment
Red blood cells	Normochromic normocytic, mildly hypochromic microcytic with acanthocytes and few elliptical cells; a few polychromatic and fragmented cells were observed with moderate Rouleaux formation.	A reactive picture with severe anemia was suggestive of microangiopathic hemolytic anemia.
White blood cells	Mild polymorphonuclear leukocytosis with neutrophils showed a left shift with toxic changes; occasional myelocytes and hyper-segmented neutrophils were noted, but there were no abnormal cells.	
Platelets	Low	

After discussing this with the consultant from the department of transfusion medicine, the patient was prepared for plasma exchange.

On the same day, he underwent his third hemodialysis session, and since plasma exchange could not be arranged immediately, he was given one pint of cryoprecipitate, four pints of fresh frozen plasma (FFP), and two pints of blood. However, there was no overall improvement in his clinical picture.

On day four, he was again transfused with one pint of blood, as his Hb levels were 6.8 g/dl. On the same day after pre-procedure preparation for plasma exchange was completed, he underwent his first cycle of therapeutic plasma exchange with 100% replacement with FFP. The post-procedural blood investigations revealed that the machine-generated platelet count was 112×10^3/µL but with a considerable improvement of the manual platelet count to 62×10^3/µL (Table 1).

On day five, he underwent his second cycle of therapeutic plasma exchange. Two pints of blood were transfused before and during the procedure. Following the second TPE, a significant improvement in the manual platelet count was observed, with it rising to 100×10^3/µL (Table 1). Later, on the same day, the fourth cycle of hemodialysis was also performed with one pint of leukoreduced blood transfusion.

After the second cycle of plasma exchange, his visual platelet count was observed to be rising above 150 with drastic clinical improvement, and he was started on 60 mg of prednisolone mane, 75 mg of aspirin, and 5 mg of folic acid daily. Further therapeutic plasma exchange was withheld on the advice of the transfusion medicine team.

He underwent 12 cycles of hemodialysis over one month. After completing 12 cycles of hemodialysis, his dialysis catheter was removed, and the patient was discharged on day 28 with a tapered dose of prednisolone while producing 2,900 ml of urine per 24 hours, with the serum creatinine dropping to 176 µmol/L.

## Discussion

Globally, 1.8 to 2.7 million cases of envenoming are reported each year [2]. According to the Annual Health Bulletin, 30,046 cases of snakebites have been reported in Sri Lanka in the year 2020, with a total of 45 deaths due to snakebite complications. Hump-nosed viper bites account for 27% to 77% of these venomous snakebites [1].

Hump-nosed vipers are of the genus *Hypnale*, and it consists of three species: *Hypnale hypnale*, *Hypnale zara*, and *Hypnale nepa*, with the latter two species being endemic to Sri Lanka [3]. The hump-nosed viper envenomation can result in local and systemic hematological, neurological, renal, pulmonary, and cardiac complications [1]. Venom-induced consumption coagulopathy is an entity that occurs due to activation of the coagulation cascade by the prothrombotic agents in the snake venom that subsequently results in consumption coagulopathy [4]. A proportion of patients who develop VICC may also manifest features of thrombotic microangiopathy (TMA) [5]. Thrombotic microangiopathy consists of a group of disorders characterized by microangiopathic hemolytic anemia, subsequent thrombocytopenia, and tissue injury caused by the occlusion of small vessels due to the formation of microthrombi [6].

Thrombotic thrombocytopenic purpura falls under the category of TMA caused by a deficiency of ADAMTS13, a plasma protease that is responsible for the cleavage of von Willebrand factor (vWF). This results in large multimers of vWF that ultimately lead to platelet aggregation, thrombosis, and subsequent thrombocytopenia due to platelet consumption [7]. Full-blown TTP is characterized by a pentad of symptoms, namely, microangiopathic hemolytic anemia, thrombocytopenia, neurologic abnormalities, kidney disease, and fever. However, the coagulation profile (prothrombin time test (PT), INR, and activated partial thromboplastin time (APTT)) is usually not altered. Even though the diagnosis can be confirmed by a demonstration of severe ADAMTS13 deficiency of <10%, due to the scarce availability of the test, in clinical practice, clinical prediction scores for severe ADAMTS13 deficiency like the PLASMIC score are used in the diagnosis of TTP [8].

The PLASMIC score takes seven components into account: evidence of hemolysis, platelet count, serum creatinine level, INR value, MCV value, active cancer, and history of a solid organ or stem cell transplant. It categorizes the risk as low-risk (0-four), intermediate-risk (five), and high-risk (six to seven) [8]. The PLASMIC score calculated for our patient was 6/7, categorizing him as a high-risk patient with an ADAMTS13 deficiency.

In our patient, even though the anemia with high lactate dehydrogenase (LDH) and fragmented cells in the blood picture were suggestive of microangiopathic hemolytic anemia (MAHA), nudging us toward the consideration of TTP as the diagnosis, the platelet count only being marginally low was a confounding factor. However, the manual platelet count revealed overt thrombocytopenia, supporting the diagnosis of TTP. The discordance between the automated platelet count and the manual platelet count could possibly be attributed to a well-known machine result where the fragmented red blood cells are counted as platelets by the machine.

An AKI was another complication encountered by our patient, requiring him to undergo hemodialysis. Acute kidney injury can be a manifestation of direct kidney injury due to snake venom nephrotoxins themselves or as a complication of TTP [9]. In our patient, the exact etiology of AKI was difficult to determine, but it was concluded to have occurred due to the cumulative effect of all the risk factors.

The currently available antivenom in Sri Lanka is not effective against hump-nosed viper bites [3], directing our management principles towards symptomatic treatment and management of complications. Meanwhile, the cornerstones of the management of TTP are plasma exchange and the initiation of corticosteroids [6].

## Conclusions

When a patient presents to a hospital following a snakebite, active surveillance for possible complications should be done, and each complication should be identified and attended to urgently and simultaneously. Thrombotic thrombocytopenic purpura is a very rare complication of hump-nosed snakebite, and if suspected, multidisciplinary management involving the physician, hematologist, and transfusion physician should be done. Plasma exchange is the cornerstone of the management of TTP, and early commencement will result in a better outcome for the patient.

## References

[1] (2023). Thrombotic thrombocytopenic purpura (TTP): practice essentials, background, pathophysiology. https://emedicine.medscape.com/article/206598-overview.

[2] Puthra S, Pirasath S, Hemal Sugathapala AG, Gnanathasan A (2020). Thrombotic microangiopathy following hump-nosed viper 'Hypnale' envenomation. SAGE Open Med Case Rep.

[3] Shivanthan MC, Yudhishdran J, Navinan R, Rajapakse S (2014). Hump-nosed viper bite: an important but under-recognized cause of systemic envenoming. J Venom Anim Toxins Incl Trop Dis.

[4] (2023). Snakebite envenoming. https://www.who.int/news-room/fact-sheets/detail/snakebite-envenoming.

[5] Rathnayaka RM, Ranathunga PE, Kularatne SA (2021). Clinico-epidemiology of Hypnale zara (hump-nosed pit viper) envenoming in Sri Lanka. Trans R Soc Trop Med Hyg.

[6] Noutsos T, Currie BJ, Wijewickrama ES, Isbister GK (2022). Snakebite associated thrombotic microangiopathy and recommendations for clinical practice. Toxins (Basel).

[7] Arnold DM, Patriquin CJ, Nazy I (2017). Thrombotic microangiopathies: a general approach to diagnosis and management. CMAJ.

[8] Chiasakul T, Cuker A (2018). Clinical and laboratory diagnosis of TTP: an integrated approach. Hematology Am Soc Hematol Educ Program.

[9] Rathnayaka RM, Ranathunga PE, Kularatne SA (2019). Kidney injury following envenoming by hump-nosed pit viper (genus: Hypnale) in Sri Lanka: proven and probable cases. Trans R Soc Trop Med Hyg.

